# Progress of the Cholera

**Published:** 1848-12

**Authors:** 


### PROGRESS OF THE CHOLERA.

The spasmodic or Asiatic Cholera, continues to be the great theme of discussion in our European exchange journals, and as it may, like former epidemics, extend to this continent, the subject, in its various bearings, is one of much interest to us also. That it will make its appearance again in America, at no very distant period, is rendered highly probable by its past history and present course. Formerly, it seemed for a long while endemic to parts of Asia ; and even when it advanced to the shores of the Caspian Sea, it was for several years stayed in its progress before invading Europe, when it swept over the continent and British Isles, and soon declared itself on this side of the Atlantic ; first in Canada, and then speedily in various parts of the United States. The present epidemic seems to be pursuing, in Europe, very nearly the same course as the former. On that occasion, its first appearance in England was on the eastern coast, at a place called Sunderland, in August, 1831; it made but little progress, however, until the early part of the following year, (1832,) when it prevailed at various parts throughout the north of England, and in London, and by midsummer, also in Edinburg, Glasgow, &c., in Scotland, as well as in the principal cities in Ireland. In Paris, it was first announced in April. In June, it appeared at Montreal and Quebec, in Canada; in July, at New York; in August and September, at Philadelphia, Baltimore, and Washington; and in October it reached Cincinnati and New Orleans.

We present this rapid sketch of the course of the disease on that occasion, that our readers may compare it with what is occurring at the present time.

From the London Medical Times of the 4th ultimo, (Nov. 1848,) we derive the following items:

In the East, at various places, the disease has not only been extremely prevalent, but attended with great mortality; thus, at Smyrna, of 3,212 attacked, 2,194 died.

*Turkey.*—The accounts received from Constantinople by the last mail, announce that the cholera is gaining ground, the daily mortality averaging nearly two hundred. The bazaars are nearly deserted.

In numerous other towns its ravages have been frightful.

*Greece.*—In the various towns and villages of Greece, the disease has prevailed but slightly, and without great mortality. Athens seems to have escaped altogether.

*Syria and Palestine.*—Aleppo, Antioch, Tripoli, Latakia, Homo, Hamah, Beyrout, Sidon, Damascus, and Scanderoon, have been visited with unusual violence by the epidemic. At Damascus, the deaths are reported as varying from 500 to 600 daily. Aleppo averages 150; Antioch about 50 daily. However, it appears to be on the decrease.

In London, notwithstanding the prevalence of scarlatina, in a severe form, the mortality, at the last dates, was less than usual; a circumstance which has frequently been observed in other places during epidemics. Ireland, so far, appears to have escaped. The following, taken from Wilmer and Smith's Liverpool Times, of the 11th ultimo, is the latest intelligence of the progress of the disease.

The Cholera.—The aggregate returns begin to look formidable. In London and its vicinity the deaths reported last week were 65; while the number of fresh cases reported daily varies between 10 and 20; and as far as we can judge at present, the mortality will be in that district about the same as last week. The general health is now 39 below the weekly average of 1847 and the four preceding years.

Reports from all the provinces are now collected—and we are happy to state that they are quite inconsiderable compared with the population. Near Hounslow, on the 8th instant, there were four fatal cases, and at Blyth six, two of which have been fatal. The remaining three on that day have occurred in Essex and Sunderland, but all the nine cases, except one, seemed to have proved fatal. It is, however, in Scotland, where the disease still commits the greatest ravages. No fewer than 468 cases have occurred in Edinburgh and the vicinity up to the 8th instant, of which 243 proved fatal, 54 recovered, while 171 were under treatment, or the result not stated. On the 8th instant only 27 new cases were reported, while there were 49 on the 7th.

At present the great manufacturing towns and districts have escaped the scourge. The malady, however, has appeared on the northern coast of France, at Dunkirk.

